# On the antiquity of language: the reinterpretation of Neandertal linguistic capacities and its consequences

**DOI:** 10.3389/fpsyg.2013.00397

**Published:** 2013-07-05

**Authors:** Dan Dediu, Stephen C. Levinson

**Affiliations:** ^1^Language and Genetics Department, Max Planck Institute for PsycholinguisticsNijmegen, Netherlands; ^2^Donders Institute for Brain, Cognition and Behaviour, Radboud University NijmegenNijmegen, Netherlands; ^3^Language and Cognition Department, Max Planck Institute for PsycholinguisticsNijmegen, Netherlands

**Keywords:** language evolution, human evolution, language contact, genetic admixture

## Abstract

It is usually assumed that modern language is a recent phenomenon, coinciding with the emergence of modern humans themselves. Many assume as well that this is the result of a single, sudden mutation giving rise to the full “modern package.” However, we argue here that recognizably modern language is likely an ancient feature of our genus pre-dating at least the common ancestor of modern humans and Neandertals about half a million years ago. To this end, we adduce a broad range of evidence from linguistics, genetics, paleontology, and archaeology clearly suggesting that Neandertals shared with us something like modern speech and language. This reassessment of the antiquity of modern language, from the usually quoted 50,000–100,000 years to half a million years, has profound consequences for our understanding of our own evolution in general and especially for the sciences of speech and language. As such, it argues against a saltationist scenario for the evolution of language, and toward a gradual process of culture-gene co-evolution extending to the present day. Another consequence is that the present-day linguistic diversity might better reflect the properties of the design space for language and not just the vagaries of history, and could also contain traces of the languages spoken by other human forms such as the Neandertals.

## Introduction

This paper argues for a much greater antiquity of human language than has normally been assumed in the language sciences. Why should researchers in the language sciences care what happened in deep prehistory? We argue here that it makes a substantial difference to how we think about language within the different disciplines that study it. We believe that the recognition that modern language has such a relatively deep antiquity ought to have the same impact on the language sciences that Lyell (1830) demonstration of the antiquity of geological process had on geology, paleontology and evolutionary theory. For example, it changes how we conceive of the biological basis for language, as a matter of the rather slow adaptation of multiple factors rather than as a saltational mutation in one or a few genes. It might also make a difference to how we think about the cultural evolution of linguistic diversity, allowing that it was slowly generated in distinct areas of the Old World followed by cross-fertilization. And just as Lyell insisted on the uniformitarian principle, whereby causal factors operative in deep time must be assumed to be currently ongoing, so linguistic and cognitive evolution must still be underway, which has a potentially profound impact on how we think about language (Levinson, 2012).

In this paper we briefly review several recent lines of evidence concerning Neandertal language and speech capacity, aiming to dispel the idea—still held in some influential circles—that the Neandertals were an inarticulate not quite human species, arguing instead that they were probably not very different biologically or cognitively from us, and that their linguistic capacities were closely similar to our own^1^. We propose that essentially modern language is phylogenetically quite old, being already present in the common ancestor of these two lineages about half a million years ago (that is, five to ten times older than is often assumed). The evidence is necessarily circumstantial, but collectively convincing we believe. Moreover, we suggest that present-day linguistic diversity might have been shaped by interactions with such archaic humans during modern human expansions across the world. We end by sketching the consequences of our proposals for language and propose a set of predictions and methods for testing them.

Several proposals about language origins make the assumption that modern language is relatively recent, arising circa 50–100,000 years ago (e.g., Bickerton, 1990, 2002; Mithen, 2005; Chomsky, 2010, 2012; Berwick et al., 2013). Several lines of evidence have been thought to suggest that Neandertals lacked language as we know it, using instead perhaps some form of protolanguage. First, general anatomical differences were suggestive of considerable evolutionary distance from modern humans, with Neandertal robustness taken to imply strength compensating for restricted intelligence. Second, early efforts to extract and analyze ancient DNA focused on mitochondrial DNA and seemed to point to significant differences between the modern and Neandertal genomes, suggesting they were a different species. Third, the recovery of parts of the fossil vocal tract and auditory system was taken to suggest important differences between Neandertal and modern human speech capacities. Fourth, there seems to be a large gap between the cultural products of Neandertals and their contemporaneous modern humans that might be accounted for in terms of a linguistic deficit. Putting these sources of information together has suggested to many influential observers (such as Noam Chomsky, Derek Bickerton and Philip Lieberman) that Neandertals lacked the specialized speech machinery, the higher language adaptations that would have gone with it, and the general cognitive flexibility (e.g., recursive thought) to make good use of language.

The suggestion has consequently been that Neandertal language, if any, had properties far too primitive to lie within the range of modern human languages. As a recent example,Chomsky (2010: 58–59) reads the evidence to show that “roughly 100,000+ years ago, the first question [why are there any languages at all? DD&SCL] did not arise, because there were no languages” and therefore that in our species alone “a rewiring of the brain took place in some individual, call him Prometheus, yielding the operation of unbounded Merge, applying to concepts with intricate (and little understood) properties….”

However, we believe that the currently available data is more consistent with a gradualist scenario, where the accumulation of small changes (both genetic and cultural) across deep evolutionary time has resulted in language and speech as we know them. Before the last common ancestor of modern humans and Neandertals, this evolutionary process may already have resulted in forms of language and speech very similar to what we presently witness, but evolution did not stop there. On both human lineages changes have continued to accumulate resulting in the modern form of language we possess today on the one hand, and something else—about which we can only speculate but which was most probably not too different—in Neandertals. To clarify: here, we understand language as the full suite of abilities to map sound to meaning, including the infrastructure that supports it (vocal anatomy, neurocognition, ethology of communication)—FLB or “faculty of language broad” in the sense of Hauser et al., 2002,^2^. Our proposals are neutral to any special role for FLN (“faculty of language narrow”) or a specifically syntactic unification ability, but [unlike Hauser et al. (2002)] we believe many detailed features of FLB are in fact unique to humans and evolved over this great timescale. Thus we attribute to Neandertals modern speech, double-articulation (separated phonology and lexicon), some systematic means of word combination (syntax), a correlated mapping to meaning, and usage principles (pragmatics).

Before we proceed we must warn that the literature we attempt to review crosses many fields and is complex, and moreover in continuous flux with the result that there are very few points of full consensus. Readers should understand that this is highly contested terrain, where each data point gives rise to conflicting interpretations, and we have restricted space here. Thus, we do not attempt to offer a comprehensive review but rather aim to highlight those aspects which are either little known to those in the language sciences, rather new, or that have been relatively neglected in discussions of language evolution, and which all favor the proposal of a greater antiquity for language. Nevertheless, we also try to mention alternative interpretations and conflicting evidence where feasible.

## The similarities and differences between neandertals and modern humans

### The evolutionary context

**Figure 1:**
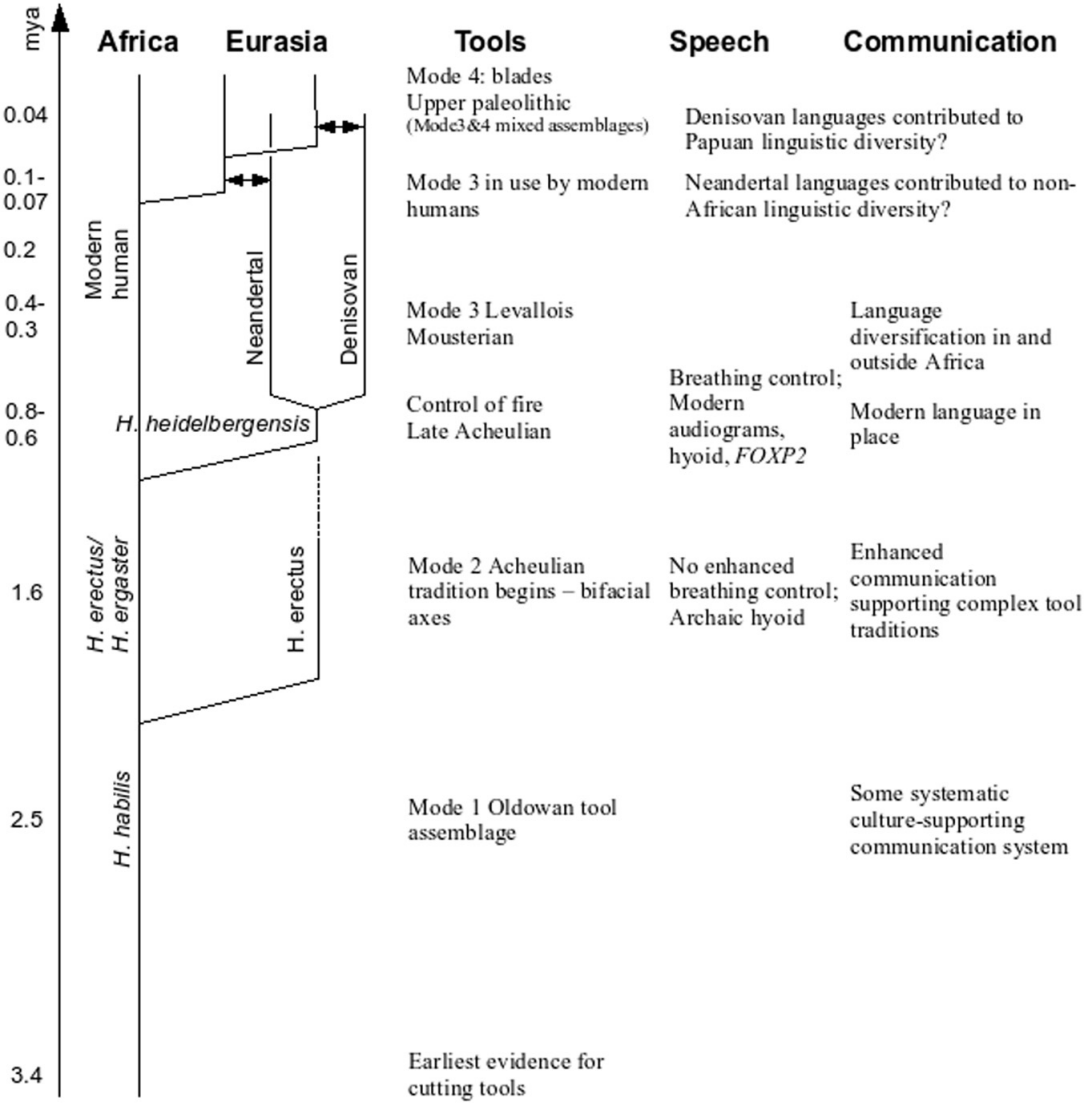
**A graphical summary of our proposal**. Dates, lineage names, and genealogical relationships between them are tentative. “Tools” lists the main toolkits in use, “Speech” describes the main evidence for advanced vocal capacities and “Communication” describes the inferred communication systems and their properties, as argued in the paper. The arrows represent admixture.

The dominant current story (e.g., Klein, 2009) in a simplified form is that, following a split about 6 million years ago (mya) from our nearest living cousins the chimps, a stone tool making hominid, *Homo habilis*, had evolved in East Africa by about 2.4 mya. By 1.8 mya, a more advanced presumed descendant, *Homo erectus*, is attested also in East Africa, a species that developed the distinctive bifacial hand axe (Mode 2 tools), and that dispersed relatively rapidly across the Old World, from England to Georgia to China and Indonesia. In Africa *H. erectus* evolved into *H. ergaster* (for some, just an African version of *H. erectus*) who evolved later into *H. heidelbergensis*, the presumed common ancestor of Neandertals and modern humans^3^. *H. heidelbergensis* and immediate successors were adept tool users, likely fashioned aerodynamic javelins, brought down large game, possibly used red ochre presumably for symbolic purposes, were regular fire users and perhaps buried the dead. They dispersed throughout Western Europe and the bulk of skeletal material comes from Atapuerca in Spain, dating to ~500 thousand years ago (kya)^4^. See Figure 1 for a sketch of these developments.

After their split from this common ancestor, the Neandertal and modern human lineages continued to diverge probably with minimal contact due to the very different and geographically distant areas which they inhabited (western Eurasia vs. Africa, respectively). By ~400 kya, individuals with Neandertal features, marked by overall bodily robustness and specific cranial shape, were already recognizable in Europe (Hublin, 2009). The very robust body proportions were probably due to adaptation to the glacial environments they inhabited, while the cranial typology was probably due to genetic drift (Weaver et al., 2007; Weaver, 2009). This was a period of dramatic climatic fluctuations, and the Neandertal range expanded and contracted in response, extending as far north as 55°N and as far east as South Siberia, and the Middle East in the south (Hublin, 2009; Green et al., 2010). This fluctuation of range may have been driven by a process of repeated local extinction and recolonization rather than by wholesale population movements tracking the changing habitats (Hublin and Roebroeks, 2009; Dennell et al., 2010). Meanwhile, the lineage leading to anatomically modern humans continued in East Africa, with early fossils of modern type from, e.g., Omo (~200 kya) and Herto (~160 kya) in Ethiopia. Modern (or near modern) humans then appear in the fossil record of the Middle East just over 100 kya (Klein, 2009: 476), and by 70 kya had begun their dispersal around the Old World. They overlapped with Neandertals first in the Middle East, then elsewhere, entering glacial Western Europe late, somewhere before 40 kya. The current evidence seems to point to a split of the two lineages by at least 400 kya, although there was repeated contact between Neandertal and modern human populations from at least 100 kya.

### The emerging picture from ancient DNA

Recent advances in genetics have allowed us to extract and analyze genetic material (ancient DNA, or *aDNA*) from hominin fossils (see Disotell, 2012 for a review). This rapidly developing field of research has already revolutionized our understanding of human evolution and promises to continue doing so. Therefore the following review must be taken as our interpretation, a current snapshot, of the fast accumulating evidence.

As mentioned earlier, initial findings from recovered Neandertal DNA, using only mtDNA (mitochondrial DNA), seemed to support the then current view that they did not contribute to the modern human genetic diversity (Stringer and Andrews, 1988; Mellars, 2005), either because of a total lack of interbreeding or a relatively low initial contribution later lost (Nordborg, 1998, 2004; Relethford, 2003; Serre et al., 2004; Weaver and Roseman, 2005; Hodgson and Disotell, 2008). However, later advances in Next Generation sequencing allowed the publication of complete Neandertal (Green et al., 2010; pre-publication release on 19 March 2013 http://www.eva.mpg.de/neandertal/press-release.html) and Denisovan (Reich et al., 2010; Meyer et al., 2012) genomes, and, as foreseen by some (Nordborg, 1998, 2004; Relethford, 2001, 2003), these revealed a much more complex story of interconnected genetic histories between three ancient human lineages.

It was found that non-African living humans carry more shared derived alleles with Neandertals than Africans do (Green et al., 2010), which was interpreted as suggesting that Neandertals and modern humans interbred (admixture estimated at 1–4%, most probably about 2.5%) during the latter's exodus from Africa, probably somewhere in the Middle East (other interpretations were also offered; Hawks, 2010; Hodgson et al., 2010). More recent work (Meyer et al., 2012; Wall et al., 2013) found that this Neandertal contribution was not equally distributed across all non-African modern humans, with about 20–40% less admixture in Europeans (estimated 6.4%) than in East Asians (9.6%), pointing to more complex admixture scenarios with at least two separate episodes or a protracted, low intensity interaction over tens of thousands of years. Alternative proposals explaining these patterns as resulting from modern human population differentiation instead of admixture (e.g., Eriksson and Manica, 2012) have been rejected (Sankararaman et al., 2012; Wall et al., 2013). The most likely period in which these interbreeding events took place is between 47 and 65 kya. An equally interesting pattern has been found for Denisovans (a sister group to the Neandertals identified only through their DNA), namely that they contributed (~4% besides the Neandertal contribution) to the present-day Papuans, Melanesians and aboriginal Australians (Reich et al., 2010; Meyer et al., 2012). Supporting these patterns of successful admixture is the finding that modern humans, Neandertals and Denisovans share a karyotype with 23 pairs of chromosomes as opposed to the other great apes which have 24 (Meyer et al., 2012).

Dating of the splits between these three ancient lineages (Denisovans, Neandertals and modern humans) involves large uncertainties due to the incompleteness of the fossil record, uncertainties in estimating mutation rates, and the difference between population (younger) and genetic (older) divergences. Estimates have been on the order of 170–700 kya for the Denisovan-modern human population split (Meyer et al., 2012) and 270–440 kya for the Neandertal-modern human split (Green et al., 2010), but recent reassessments of mutation rates suggest, e.g., 420 to 780 k for the latter (Hawks, 2012).

Thus, aDNA suggests that our evolutionary history is far from a simple and continuous progression of a single lineage leading to us, but instead reflects a reticulated history, involving at least three closely related branches that exchanged genes, probably repeatedly. One consequence is that we should probably stop thinking about these three lineages as separate species [in the sense of Mayr's (1942) Biological Species Concept where interbreeding is definitional, but see Hey (2001) for many alternative definitions]. Indeed it seems likely that further ancient hominins will turn up, further complicating this reticulated history. More interesting than the species question is how much interbreeding actually took place and under which scenario, with some model-based estimates suggesting that these might have been very rare events of less than 2% (and as low as 0.5% in some scenarios) successful matings (Currat and Excoffier, 2011) or 1 mating in 12–77 generations (Neves and Serva, 2012). However, more work is needed given various complicating factors such as differences in population size and later demographic events. It is important to appreciate that different Neandertal genes are found in different modern human individuals, which “suggests that the number of contacts was not very small—more like the low thousands or high hundreds than dozens” (Hawks, 2013). Whatever the rates of interbreeding, the genes acquired by modern humans may have been crucial. For it is possible that some Neandertal and Denisovan genes conferred strong selective advantages in the out-of-Africa environment, especially in the immune system, and have very high frequencies in modern populations despite low levels of interbreeding (Hawks and Cochran, 2006). Proposals include immune system genes such as in the HLA system (Abi-Rached et al., 2011), the *STAT2* gene (Mendez et al., 2012) and the *OAS* gene cluster (Mendez et al., 2013b).

More important, however, is what the direct comparisons between the Neandertal, Denisovan and modern genomes can tell us about their similarities and differences. As expected given their recent common ancestry and their successful admixture, these three genomes are extremely similar, sharing the vast majority of innovations since the split from chimps (Green et al., 2010; Meyer et al., 2012), such as ~91% of the “human accelerated regions” (HARs)—parts of the genome that changed since that split, but are otherwise very stable throughout vertebrates. Potentially relevant for language and speech, they share for example the same “human specific” two amino-acid substitutions in *FOXP2* (Krause et al., 2007), the best-known gene hitherto linked to language, lending support to our hypothesis that Neadertals were language users (Trinkaus, 2007).

Nevertheless, there are subtle differences between the genomes of the lineages: while the *FOXP2* exons (the protein-coding sequences) are identical, recently Maricic et al. (2013) have reported that a regulatory element within intron 8 of *FOXP2* binding the *POU3F2* transcription factor differs between Neandertals and modern humans and might have been the target of recent positive selection since their split (Ptak et al., 2009). However, it is currently unclear what effects this change has and, importantly, the ancestral (“Neandertal”) allele is still present at ~10% frequency in present-day Africans (Maricic et al., 2013), showing that this variant is well within the modern human variation^5^. Besides genes affecting the skin (*TRPM1, HPS5*), the eye (*RP1L1, GGCX*), metabolism (*THADA*), the skeleton (*RUNX2*), and dentition (*EVC2*), some genes affecting the brain and nervous system are also different between us, on the one hand, and the Neandertals and Denisovans on the other (Green et al., 2010; Meyer et al., 2012). A recent review (Somel et al., 2013) lists several such genes including the intron 8 of *FOXP2* and a protein change in *CNTNAP2*^6^ (another gene implicated in speech and language; Vernes et al., 2008; Fisher and Scharff, 2009), *MCPH1* and *ASPM* (brain development; Cox et al., 2006), the dopamine receptor gene *DRD5*, and *MEF2A*, a gene perhaps involved in prolonged developmental plasticity of the brain.

Taken together, these suggest that Neandertals, Denisovans and modern humans were very similar, although of course not identical, hominins. More research will help clarify what these small differences entail, and to what extent they are within the range of modern human variation, but we would propose that they are likely to be quantitative in nature. It is important, however, to keep in mind that the human genome is very complex and that regulatory changes can have hard-to-predict effects, making any inferences from the identity (or not) of gene sequences to speech and language necessarily tentative. Nevertheless, the genetic story so far suggests that Neandertals and Denisovans had the basic genetic underpinnings for recognizably modern language and speech, but it is possible that modern humans may outstrip them in some parameters (perhaps range of speech sounds or rapidity of speech, complexity of syntax, size of vocabularies, or the like).

### The skeletal morphology

Neandertals are considered to form a relatively coherent group of fossils. Morphologically, they are characterized by cranial differences from modern humans and by post-cranial robustness (Klein, 2009). The cranial differences involve long and low braincases with volume comparable to (even exceeding) that of modern humans, and the face was prognathous with projecting dentition, and chinless. Many minor details, such as dentition and angle of the semicircular canals, are distinctive. The rest of the differences amount to very robust upper limbs, revealing remarkable muscular strength, and general stockiness reflecting adaptation to the cold climates they inhabited. The robustness may be a reflection of recurrent bio-mechanical loading (and thus cultural differences in life style) as much as of genetic differences (Klein, 2009: 461), with the cranial morphology as mentioned most probably a result of genetic drift (Weaver et al., 2007; Weaver, 2009).

These differences between modern humans and Neandertals increased over time, and the “classic” Neandertal phenotype is found between 190 and 30 kya. Nevertheless, there may be some intermediate fossils, possibly reflecting the interbreeding that we now know to have occurred through the DNA evidence reviewed above. Probably the best-known case is represented by the child discovered at the Abrigo do Lagar Velho, Portugal (Duarte et al., 1999), which has been interpreted as a Neandertal-modern human hybrid dating from about 24 kya (Duarte et al., 1999; Trinkaus and Zilhão, 2003), an interpretation apparently supported by the recent analysis of its pattern of dental development (Bayle et al., 2010)^7^. Given the burial context, it has been argued that the child had been accepted as a full member of the community speculating that this type of admixture was viewed as tolerable at least, and was frequent enough to gain social acceptance (Zilhão and Trinkaus, 2003). However, this interpretation is hotly contested (e.g., Tattersall and Schwartz, 1999) and must be taken as speculative. Other remains which have been suggested to show signs of dual ancestry (Trinkaus, 2007) include the European early modern humans from Peştera cu Oase (Trinkaus et al., 2003; Rougier et al., 2007), Peştera Muierii (Soficaru et al., 2006), Mladeć (Teschler-Nicola, 2006), and Riparo Mezzena, Italy (Condemi et al., 2013), but these interpretations are far from being generally accepted.

Before the recent DNA evidence became available, there was no consensus as to whether these gross physical differences were enough to presume that modern humans and Neandertals constituted different species (see Dediu, 2007). This reflects the many distinct notions of species in biology (Hey, 2001, lists 24 definitions), and in part it stems from different animal models used. Interbreeding might be taken as evidence than modern humans, Neandertals and Denisovans all belonged to one biological species, but introgression (back-crossing of fertile hybrids with a parent species) does occur across species boundaries (Mallet, 2005).

### Neandertal infant maturation

One important (but contentious) area concerns the developmental schedule for maturation of Neandertal infants. Modern human infants develop slowly after birth, resulting in a dependency during the first years of life crucial for the learning of language and other aspects of culture. The developmental trajectory in turn depends on the size of the birth canal: if small, infants will be more dependent and birthing will be difficult, suggesting “obligate midwifery and all of the attendant social implications” (Franciscus, 2009: 9126).

Weaver and Hublin (2009) report the reconstruction of a Neandertal birth canal and conclude that there are some differences in the orientation of the neonate during birth, but that the pelvic area of humans and Neandertals was very similar and that “a human-sized neonate would have been able to pass through Tabun's birth canal” (p.8154). Likewise, the neonate brain size was similar to that of modern humans (de León et al., 2008), but the developmental trajectory seems to have been relatively different (Gunz et al., 2010). Recent evidence (Lalueza-Fox et al., 2010) from a Neandertal family assemblage was interpreted as indicating an interval of about 3 years between consecutive births, similar to that reported for modern hunter-gatherer groups. This suggests that the Neandertal life history was as slow, or even slightly slower, than that of modern humans, with the origin of this pattern predating the last common ancestor of these lineages. This inference seems supported, among others, by the analysis (de Castro et al., 1999, 2010) of the dental eruption pattern shown by a mandible attributed to *Homo antecessor* (0.8–0.96 mya) suggesting that a prolonged childhood similar to that of modern humans is a relatively early characteristic of the genus *Homo*.

We may expect further insight into these issues as we come to understand what the differences between modern and Neandertal DNA imply functionally (section The Emerging Picture from Ancient DNA): for example *MEF2A* has recently been suggested to be involved in extending brain plasticity in our lineage and underwent a regulatory change in the last 0.5m years (Somel et al., 2013). Therefore, it is possible that this could imply a somewhat different developmental trajectory and gene expression in the prefrontal cortex with a more limited period for the acquisition of a complex learned communication system in Neandertals.

Thus, the evidence so far seems to point to a similar but not identical pattern of birth and slow development in Neandertals and modern humans, capable of supporting the cultural transmission required for complex language and culture. Moreover, it seems highly probable that hybrids resulting from mixed mating would have been able to be born by mothers of any lineage and would have been capable of normal development.

## Neandertal speech, language and culture

### Speech and hearing

Fossilized parts of the vocal and auditory anatomy provide important information about ancient capacities for speech production and perception. In principle, combined with appropriate models, they could allow relatively robust inferences concerning the capabilities of past humans. But, in practice, it turns out that there is enough latitude for fierce debates concerning the appropriateness of the models used and their capacity to distinguish competing hypotheses (Fitch, 2009a).

In species with elaborate conspecific communication systems, there tends to be a precise match between the broadcast bandwidth and the tuning of perceptual acuity (see, e.g., Lafon, 1968; Kojima, 1990). The possession of articulate speech therefore implies that both production and perception are attuned to each other, so that parameters carrying the bulk of the speech information are optimized in both production and reception.

Human audiograms differ from those of other living primates by showing higher sensitivity in the 1–6 kHz range and especially between 2 and 4 kHz, just where chimpanzees show a relative loss in sensitivity (Martínez et al., 2004, 2008). Using 3D CT-scans of five fossil individuals from the Sima de los Huesos site in Spain, Martínez et al. (2004) reconstructed the anatomy of their external and middle ear, to which they further applied an electrical circuits model of the sound power transmission through these structures. The results suggest that these fossil hominids had a modern human-like pattern of sound perception, which clearly differs from the chimpanzee pattern in the region around 4 kHz (Martínez et al., 2004, 2008), strongly supporting the inference that their hearing apparatus could support modern speech perception (for a more skeptical position, see Fitch, 2010: 325). By the principle of the matching of broadcast and perception bandwidths, we can presume that speech was produced in the current auditory range. The interesting twist is that these fossils are attributed to *Homo heidelbergensis* and date from approximately 500 kya, that is around or shortly after the time that the lineages of Neandertals and modern humans may have initially separated. Even if these fossils belong to the evolutionary lineage leading not to modern humans but to Neandertals, the date nevertheless suggests that modern audition almost certainly predates the common origin of these two lineages.

Quam and Rak (2008) have recently described and analyzed a new set of Neandertal and modern human ear ossicles from Qafzeh and Amud which date 50–100 kya. They conclude that the range of morphological variation in the Neandertal ear bones is included within the modern human range and that what may differ are the relative frequencies of these variants in the two populations. Therefore, it can be safely concluded that Neandertal ear ossicles are essentially modern, further supporting the idea that their audition was very similar, if not identical, with that of modern humans.

On the production side, there has been considerable controversy focused on the descent of the larynx and the role of the hyoid bone. Nevertheless, we think it is clear that the number and diversity of clues, taken together, clearly point in the direction of a modern capacity for speech in the common ancestor of Neandertals and modern humans. Neandertal hyoids are essentially modern (from Kebara, Israel; Arensburg et al., 1989, 1990; from El Sidrón, Spain; Rodríguez et al., 2003, and from Sima de los Huesos, Spain—from Homo heidelbergensis; Martínez et al., 2008). The modernity of the Neandertal hyoid contrasts markedly to the archaic characteristics of the *Homo erectus* specimen from Castel di Guido, Italy (Capasso et al., 2008) and of the *Australopithecus afarensis* specimen from Dikika, Ethiopia (Alemseged et al., 2006). The morphology of the hyoid bone is intimately connected to the issue of air sacs, present in many primate species (de Boer, 2009): these are cavities filled with air and connected to the vocal tract (Hewitt et al., 2002). Their acoustic function is not entirely clear, but recent work (de Boer, 2009) seems to suggest that their presence has deleterious effects on speech by reducing the range of distinctive speech sounds which can be produced. Thus, given the current fossil evidence, air sacs had probably disappeared between *H. erectus* and the last common ancestor of modern humans and Neandertals.

While admitting that the Neandertal hyoid bone was essentially modern in morphology, Fitch (2009a) argues, following Laitman et al. (1990), that this is not enough to prove a modern position within the vocal tract, nor a modern capacity for speech. The position of the hyoid bone within the vocal tract has received a lot of attention since the proposal by Lieberman and Crelin (1971) that it can be inferred from features of the cranium (the styloid processes or basicranial angle) and that this position can tell us something about the vocal capabilities of fossil hominids. However, as extensively shown by subsequent work (Falk, 1975; Le May, 1975; DuBrul, 1977; Houghton, 1993; Fitch, 2009a), the position of the hyoid cannot be safely inferred from the skeletal features suggested and the high position of the Neandertal hyoid proposed by Lieberman and Crelin (1971) cannot be justified, with a lower position being, in fact, much more probable. Moreover, as demonstrated by Fitch (2000, 2009a), many mammals can dynamically lower their larynx during vocalizations, implying that the “rest” position is not necessarily a good indicator of the dynamic vocal capabilities. A further complication is added by the fact that Boë et al. (2002) have claimed, using an articulatory model, that the vowel space of Neandertals with a high hyoid was comparable to that of modern humans; but these results have been recently challenged (de Boer and Fitch, 2010) on grounds of circularity, as they were using a model developed on modern human data. Nevertheless, we concur with Fitch (2009a)'s conclusion that “the significance of the descent of the larynx […] has been overestimated” (p.133) (see also Nishimura et al., 2006).

Two other proposed fossil clues allowing inferences related to the evolution of speech concern traces of the capacity to control the tongue (the hypoglossal canal) and the respiratory muscles (the thoracic vertebral canal), respectively (Fitch, 2009a). The first clue seems to be unable to offer much information, as the size ranges in modern humans and other apes, including chimps, show substantial overlap (DeGusta et al., 1999; Jungers et al., 2003). However, the second is crucial, for voluntary control of breathing is a prerequisite for any complex speech production (MacLarnon and Hewitt, 1999), and this requires cortical control taking over from the autonomous respiratory control in the brain stem. This is achieved by extra enervation of the intercostal muscles and diaphragm, visible in fossils as an enlarged vertebral canal. The crucial evidence is that the Nariokotome boy (a well-preserved *H. erectus*) has no such enlarged canal, but both modern humans and Neandertals do, implying that the common ancestor also did. Every stage of speech production depends on this cortical control, which allows the sharp in-breath, the slow release and volume modulation required. This crucial ingredient, for which there is no likely other motivation, is thus present before 0.5 mya.

The voluntary control system is particularly relevant for the issue of vocal imitation and learning (Fitch, 2010: 350), which is normally described as rare in the primate order (but see, e.g., Wich et al., 2012). Direct evidence of vocal imitation in fossil hominins is of course missing, but precise tool replication provides ample evidence for the necessary cognitive capacity in another modality.

In sum, the evidence points to modern speech capacities in the common ancestor of Neandertals and modern humans. The auditory specializations for speech on the modern bandwidth are present, the morphology of the larynx looks modern, and air sacs have been replaced by a finely controlled pulmonic airstream mechanism for vocalization. In addition, the gene that is known to be involved in the fine motor control necessary for speech, *FOXP2*, has its modern form (although possibly not all of its modern regulatory environment). Interestingly, all these changes occurred in the transition from *Homo erectus* to *Homo heidelbergensis*, the common ancestor to both Neandertals and modern humans. We suggest therefore that this common ancestor was an articulate mammal.

Now, there is an old strand of speculation going back to Darwin (1871) that imagines a scenario in which speech evolved under sexual selection for “producing true musical cadences, that is in singing” [see discussion in Mithen (2005); Fitch (2009b)]. Preadaptation of speech for something other than language cannot be ruled out, but the perceptual bandwidth mirroring modern speech looks undermotivated for singing (which in modern humans is mostly concentrated at the higher end of the bandwidth used in speech). In addition, it is notable that the relevant animal models for singing as a possible precursor to language are not found among the social species; social mammals have acquired vocal learning through other routes, namely for broader social communication. Neurologists have long noted double dissociations between amusia and aphasia, i.e., loss of musical ability and loss of speech, and cognitive scientists have pointed to many ways in which the processing systems seem to be distinct (Peretz, 2006). Even the most enthusiastic proponents of processing overlap between language and music admit that music diverges in fundamental ways, such as its organization of pitch and rhythm, the absence of a categorical basis, and its lack of propositional meaning (Patel, 2008). If there was any singing precursor to language it must lie right back at the beginning of the human lineage, millions of years ago. There are moreover many other reasons to suspect language was present to utilize the speech channel, which we now turn to.

### Culture and language

The Neandertals had a complex stone tool technology (the Mousterian) that required considerable skill and training, with many variants and elaborations (see Klein, 2009: 485ff). They sometimes mined the raw materials at up to 2 meters depth (Verri et al., 2004). Their stone tools show wear indicating usage on wood, suggesting the existence of a wooden material culture with poor preservation, such as the carefully shaped javelins made ~400 kya from Germany (Thieme, 1997). Tools were hafted with pitch extracted by fire (Roebroeks and Villa, 2011). Complex tool making of the Mousterian kind involves hierarchical planning with recursive sub-stages (Stout, 2011) which activates Broca's area just as in analogous linguistic tasks (Stout and Chaminade, 2012). The chain of fifty or so actions and the motor control required to master it are not dissimilar to the complex cognition and motor control involved in language (and similarly takes months of learning to replicate by modern students)^8^. The Neandertals managed to live in hostile sub-Arctic conditions (Stewart, 2005). They controlled fire, and in addition to game, cooked and ate starchy foods of various kinds (Henry et al., 2010; Roebroeks and Villa, 2011). They almost certainly had sewn skin clothing and some kind of footgear (Sørensen, 2009). They hunted a range of large animals, probably by collective driving, and could bring down substantial game like buffalo and mammoth (Conard and Niven, 2001; Villa and Lenoir, 2009).

Neandertals buried their dead (Pettitt, 2002), with some but contested evidence for grave offerings and indications of cannibalism (Lalueza-Fox et al., 2010). Lumps of pigment—presumably used in body decoration, and recently found applied to perforated shells (Zilhao et al., 2010)—are also found in Neandertal sites. They also looked after the infirm and the sick, as shown by healed or permanent injuries (e.g., Spikins et al., 2010), and apparently used medicinal herbs (Hardy et al., 2012). They may have made huts, bone tools, and beads, but the evidence is more scattered (Klein, 2009), and seemed to live in small family groups and practice patrilocality (Lalueza-Fox et al., 2010).

The inference of language capacity from the archaeological record is a controversial endeavor (d'Errico and Vanhaeren, 2009) and the dangers of such inferences are well illustrated by the myth of the “modern human revolution.” This is the proposal that the cultural efflorescence seen in Upper Paleolithic Europe from ~40 kya was due to a fundamental cognitive shift resulting from a sudden mutation giving rise to a new species possessing the so-called “modern package” (e.g., Dunbar, 1996; Mithen, 1996; Donald, 1999; Bickerton, 2002; Gabora, 2003). Some of the differences between Neandertals and modern humans that are often invoked concern the lack of art and personal ornaments, the absence of large-scale exchange networks or projectile weapons, the meager investment in campsites, the relatively narrow range of prey and in particular the apparent neglect of fishing (McBrearty and Brooks, 2000; Stringer, 2002; Henshilwood and Marean, 2003; Mellars, 2005; Klein, 2009; Roebroeks and Verpoorte, 2009).

However, many of these “hallmarks” of modern human behavior found in the European Upper Paleolithic turn out to be quite exceptional features of pre-Neolithic human cultures. The ethnographic records of first contact with most hunter-gatherer groups lack all these material expressions of symbolic exuberance: most symbolic activity, like language, simply does not fossilize. Nothing like the European Upper Paleolithic explosion of symbolism is found among the early colonizers of the Americas—but they were modern humans just 12,000 years ago. It is worth pointing out too that the notion of symbolism invoked in these discussions has little to do with language: the peculiarity of linguistic symbols is that they denote by abstract convention, while a cave painting of a horse denotes by iconic similarity, a principle that plays a very minor role in language. In addition, some of the apparent Neandertal failings, like lack of use of marine resources, now seem artifacts of the sites investigated earlier (see Alperson-Afil et al., 2009).

The myth of a “modern human revolution” is now rejected by archaeologists, although it lingers on in linguistic circles, as illustrated, for example, by Chomsky (2010). The myth dissolves as soon as one considers the archaeological record of the whole Old World, and especially of Africa, where a gradual, piece-meal process of cultural accretion took hundreds of thousands of years (McBrearty and Brooks, 2000). The supposed lack of signs of symbolic activity has been exploded with the recent discovery of personal ornaments and pigments at Neandertal sites (d'Errico and Soressi, 2002; d'Errico and Vanhaeren, 2009; Watts, 2009), intentional burials in fetal position, possibly with grave goods (Grün and Stringer, 2000; Klein, 2009) and other “hallmarks” of modern human behavior (Shipman, 2008; Riel-Salvatore, 2010). The case of the decorated pendants in the Arcy Chattelperronian is hotly debated (see below, Caron et al., 2011; Higham et al., 2010). But perforated shells with ochre coloring extracted from sources at some distance have recently been found in Spain, dating to about 50 kya, long before contact with modern humans in that region (Zilhao et al., 2010). In addition, on the other side of the coin, why did anatomically modern humans fail to invent the cultural assemblages they later produced in Europe earlier in the 150,000+ years leading up to their colonization of those parts? They were cohabiting with the Neandertals in the Levant for perhaps some 50,000 years, using the identical basic material culture (Klein, 2009). Part of the answer may be that the abilities were present but dormant in both lineages, awaiting a *cultural revolution* that itself spurred a demographic revolution in, and an exodus from, South and East Africa (Mellars, 2006).

The range of classic Neandertal brain sizes fully overlaps the range of modern humans (Klein, 2009: 308) and correcting for body mass highlights this similarity (Klein, 2009: 728). It is possible however that the structure of their brains might have not been identical to that of modern humans: the “occipital bun” suggests a possible development of visual areas which could point to a relatively different cognitive style (Pearce et al., 2013). If we follow Dunbar (1993), using the predictions based on neocortex sizes, even accepting the recently proposed adjustment for body size and a larger visual system (Pearce et al., 2013), Neandertals would be expected to have had large group sizes (~115), relatively similar to modern human hunter-gatherers (~144), requiring complex social systems.

Especially interesting is the late Neandertal period of contact with modern humans when there are numerous signs of cultural borrowing. The Châtelperronian (e.g., Floss, 2003) is associated with Neandertals and a clear blend of the Neandertal Mousterian technology and the modern human Aurignacian technology. The stone tool assemblages include both types, there are symbolic elements like bone beads and pendants, complex foundations for ancient huts, etc., strongly suggesting cultural diffusion of modern human technology to Neandertals (Klein, 2009: 655). Recently, some doubt has been cast on whether the personal ornaments found at Arcy-sur-Cure in Châtelperronian layers were actually made by Neandertals—some argue they must be intrusions from later modern human strata (Higham et al., 2010; Mellars, 2010), but this now seems unlikely Caron et al., 2011; Hublin et al., 2012. A similar pattern would be found in the ethnographic record of early colonial contact with indigenous peoples, namely a rapid adoption of new technology. Cognitive capacity is obviously best measured by the ability to adopt diffused technology rather than by the ability to invent it, which owes much to cultural advance.

Neandertal culture, basically identical to modern human cultures before the Upper Paleolithic innovations, seems also to fall within the spectrum of modern human cultural variation in the ethnographic record. Various modern hunter-gatherers have produced archaeological records very similar or even considerably simpler than the Neandertal ones (Roebroeks and Verpoorte, 2009), some well-known examples being the North American early Archaic (Speth, 2004) and the Tasmanians (Richerson et al., 2009), who lacked bone tools, clothing, spear throwers, fishing gear, hafted tools and probably the ability to make fire (Henrich, 2004). Recollect also the Yaghans of Tierra del Fuego whose complete nakedness in frigid conditions and absence of all but the simplest material culture so amazed Darwin: “without exception the most curious and interesting spectacle I ever beheld: I could not have believed how wide was the difference between savage and civilized man: it is greater than between a wild and domesticated animal.” (Darwin, 1845: Ch. 10, p. 216).

Like these groups of modern humans with rather simple technology, the relative cultural simplicity of Neandertals compared to European modern humans can probably be best understood in its *demographic* context. Neandertal populations of Europe had something like one tenth the population of the modern humans who immediately succeeded them (Mellars and French, 2011). In general, Neandertals had very low population densities, which coupled with the repeated local extinction and recolonization (Hublin and Roebroeks, 2009; Dennell et al., 2010; Dalén et al., 2012), would have inhibited the growth of complex technology. There are intimate relationships between demography and cultural complexity, which can be partly understood in terms of cultural niche construction, the process by which organisms can significantly alter the selective environment they inhabit (Habgood and Franklin, 2008; Powell et al., 2009; Richerson et al., 2009; Kline and Boyd, 2010). The kind of cultural and technological elaboration characteristic of the post-Neolithic is intrinsically connected to intensive population pressure, and the ecological reworking associated with it. One possible reason for the cultural limitations of small populations has to do with the transmission fidelity of culture, with only larger populations having the variance and division of labor to maintain the quality of skills (Henrich, 2004; see though Read, 2006).

However, language seems to behave in a different manner, due to its design properties which require “parity” (similarity of systems) between communicators: here, large populations erode complexity (because of the need to communicate across groups), and small ones allow (but do not require) it. Consequently, highly complex languages (with elaborate morphology and irregularity) tend to be spoken by small groups (Lupyan and Dale, 2010). From this, we might conjecture that Neandertal languages may have had more complex categories than the languages spoken by the often larger modern human groups that followed, and in particular by contemporary large-scale societies. We can speculate that they perhaps had the features typical of languages spoken in small traditional societies today: sizable phoneme inventories, complex morphosyntax, high degrees of irregularity, and vocabularies in the tens of thousands. We can also be fairly sure, due to the relatively isolated nature of the groups, that there were many distinct languages. We could even hazard the prediction on the basis of the genes they carried, that the chances are they spoke tone languages (Dediu and Ladd, 2007), as will be made clear in the discussion below. All this is speculation, but perhaps as our knowledge of the sociolinguistics of small-scale societies and of functional genetics improves, we may be able to put these guesses on a firmer basis.

It seems that speculations on human prehistory often deny cultural elaboration itself the causal role it so clearly deserves. Greater cultural elaboration must, the arguments seem to imply, depend on something else: greater intelligence, a speciation event, or some biological basis for an independent demographic spurt. But human culture is a spiral which under the right conditions will simply ratchet up. The right conditions are time left over from subsistence activities, strong norms of parental investment in the young, relative health, sufficient peer competition, ecological wealth for conspicuous consumption, etc. These enabling conditions have to be met, and then incremental cultural transmission will do the rest. There is no other way to explain the cultural diversity in the modern ethnographic world. The early modern humans that invaded Europe and eventually replaced the Neandertals had the advanced technology, just like Captain James Cook had when he arrived in Australia in 1770—Cook's advantage wasn't his smarts so much as thousands of years of accumulated technological prowess.

Thus, we believe there is no argument to be made from Neandertal culture to the absence of language. The paucity of preserved symbolic material is also observed in early modern humans, and many modern ethnographic settings. On the contrary, nothing like Neandertal culture, with its complex tool assemblages and behavioral adaptation to sub-Arctic conditions, would have been possible without recognizably modern language.

Finally, we should turn to the issue of Neandertal extinction, often presumed to be a consequence of cultural and technological failure—modern humans wiped them out, as we continue to do to so many other species, and indeed to small ethnic groups of our own species. At the present, there is no clarity on this issue. On the one hand, there were long periods of coexistence with modern humans, especially outside Western Europe (and up to 10,000 years within it, as mentioned earlier), and much evidence of cultural borrowing as reviewed. On the other hand, some redating of fossils suggests that Neandertals may have retreated from areas of Europe before modern humans ever got there, under the severe conditions of the last glaciation (OIS3; see Stewart, 2005; Wood et al., 2013). Some scholars incline to the view that Neandertal populations were absorbed rather than extinguished, hence the intermediate traits sometimes found in late Neandertals (Condemi et al., 2013). Their demography was always fragile. It is worth remembering that many modern human pioneers in difficult environments (like the Norse of Greenland) also simply failed to make it through. Perhaps the disappearance of Neandertals was due to some mix of climate change, absorption, competition and genocide.

## Consequences for the study of language and linguistics

As stated at the outset, we understand language as the full suite of abilities to map sound to meaning, including the infrastructure that supports it (vocal anatomy, neurocognition, ethology of communication, theory of mind, etc.). The assemblage of these prerequisites took place in deep time, we have argued, so that speech and language are ancient, being present in a modern-like form over half a million years ago in the common ancestor of Neandertals and modern humans, the result of evolution in the prior one million years or so as *H. heidelbergensis* evolved from *H. erectus*. If accepted, that multiplies the time depth of modern language capacities between five and tenfold over the numbers (typically 50,000 or 100,000 years) often mentioned in the literature in the language sciences.

After *H. hiedelbergensis*, biological and cultural evolution continued in each human lineage (and still goes on in present-day humans; Dediu and Ladd, 2007), one inside and one outside Africa, resulting in the accumulation of cultural, and no doubt some minor biological, differences in speech and language. Thus, when the two groups met again, during the modern human expansions out of Africa from 100 kya, we would argue that their speech and language capacities would have been comparable and compatible. We list below some of the direct consequences of this perspective.

*First*, a simplistic saltationist story, involving a point mutation, as proposed by, for example, Chomsky (2010), can no longer be supported. Instead we have to think in terms of an evolutionary trajectory where language and cognitive abilities accumulate and change, a process still ongoing to this day. Pinker and Bloom (1990) made the case 20 years ago for viewing language as a complex adaptive system that has evolved under natural selection for the purposes of communication, but their arguments seemed weak against the then proposed time-scale for language evolution in the last 50,000 years or so: how could such a complex, intricate system have evolved in a mere couple of thousand generations? The recognition of the antiquity of language removes this impediment to an evolutionary account of the emergence of modern language^9^.

Incidentally the view that language evolved over deep time does not entail (phyletic) gradualism, the idea that evolution moves at a fixed pace by successive tiny adaptations—not even Darwin (though mesmerized by Lyell's geological perspective) ever held that view. As far as language is concerned, the assemblage of the prerequisites for speech and language in the transition from *H. erectus* to *H. heidelbergensis* may well have been punctuated at times by relatively large changes in language-related features. Our reading of the current ancient DNA evidence is that the later split into the three (currently known) interbreeding lineages does not seem associated with punctuated and rapid development of many language-related genes in our own lineage in the last hundred millennia or so—but we can rely on rapid progress in this field to clarify the issues here. Thus, to hazard a prediction, we expect that most of the genetic differences between modern humans, Neandertals and Denisovans (and the yet-to-be-discovered lineages) are in terms of non-fixed shared alleles and the few that are fixed result in quantitative and relatively small differences in speech and language.

On the view advanced here, speech and language were largely co-evolving capacities and the study of speech production and comprehension ought to come back to center stage, where it has been displaced by an emphasis on syntax. For example, we need to better understand the genetic foundation for the cortical control of breathing, the tongue, the velum and the vocal folds, for this may give us better clues to the sequence of evolutionary adaptations involved. Absent from other primates, for example, is the lateral cortical system, providing direct connections between cortex and larynx (Fitch, 2010: 350). The idea that human language initially went through a sign-language or gestural language phase has become popular again in part through the discovery of mirror-neurons, offering an apparently automatic translation from manual action to action-understanding (Arbib, 2005). In addition, Call and Tomasello (2007) have cogently argued that ape gesture is connected to intentional communication while ape vocalizations are more reflex signals (as reflected in the lack of cortico-laryngeal connections). Nevertheless, any supposed phase of purely gestural communication must date back at least as far as early *H. erectus*, and thus a million or more years ago. There is no evidence whatsoever of adaptation of the hand to communicative functions, while there is ample evidence of systematic adaptation of the vocal apparatus to speech, and we have shown that this was more or less in place by half a million years ago. Modern human communication is intrinsically multimodal, using gesture and speech, or at least hand and mouth and face, as evident in any current human interaction—this appears to be a single system. The recurrent natural emergence of sign languages attests to the unified nature of a hand+mouth system, since sign languages merely shift the burden from mouth to hand but use both.

*Second*, the deep time frame supports the idea that the foundations for language were incrementally acquired. Hurford (2003), amongst many others, has tried to spell out these pre-adaptations. Early candidates would be the *cooperative instincts* (Tomasello, 2008) and the interactional ethology typical of all modern humans (Levinson's, 2006, “interaction engine”), making possible the cultural transmission of tool-making visible in the archaeological record. Universals in language usage reflecting this interactional infrastructure for communication seem considerably more invariant than language structure (Stivers et al., 2009) and evidenced in early infancy, suggesting ancient phylogeny. Crucial here is the intention recognition that underlies human communication but is separately instantiated in neurocognition (Noordzij et al., 2010). Developing a finer-graded set of distinctions in these underlying capacities will make it easier to search for precursor abilities (Haun et al., 2006). The increasingly complex speech system must have come later, with the more complex aspects of language—phonology, syntax, and lexicon—being the last to evolve. Understanding the relative roles of genetic bases and cultural elaborations in these higher levels of linguistic structuring can best be done by comparing the extant languages and finding common denominators not attributable to shared cultural ancestry, which contrary to linguistic orthodoxy are actually few and far between (Evans and Levinson, 2009; Levinson and Evans, 2010). Notice that the picture just sketched inverts the usual suppositions, which assume a genetically coded, fixed linguistic structure, with variable cultural uses—far more plausible is a slow accumulation of the genetically influenced motivations and contexts for language usage, making it possible to “outsource” much of language structure to the newly evolved capacity for culture^10^.

Although we have stressed here the relative antiquity of modern language—we have after all argued for at least a tenfold increase in time depth from the c. 50,000 years sometimes quoted (e.g., Chomsky, 2007; see also Klein, 2009: 648–649)—still, on an evolutionary timescale half a million years is a flash in the pan. It pales beside the animal models sometimes appealed to by linguists, like echolocation in bats (Teeling et al., 2005) or song in passerine birds (Christidis et al., 2002) which both have origins over 50 mya. Language as we know it must then have originated within the ~1 million years between *H. erectus* and the common ancestor of Neandertals and us. That is still a remarkably short period to evolve a complex system and the implication must be that language abilities were relatively rapidly cobbled together from preadapted cognitive and neurophysiological structures.

A *third* possible consequence is that there may be ample scope for the interplay between population genetics and linguistic diversification. Dediu and Ladd (2007) showed an association between the frequency of certain genes involved in brain growth and development (*ASPM* and *Microcephalin*) and the prevalence of tone languages, suggesting that slight differences in population genetics can act as cultural “attractors,” making it slightly more likely that certain linguistic types will propagate (Dediu, 2011). Since the variants of these genes associated with non-tonal languages seem to have been absent from Neandertals^11^, it is reasonable to assume that Neandertal languages were most probably tonal. Such genetic biases would generally act as cultural attractors over relatively large time periods, so the increased time-window for language history suggests that there may be significant numbers of such effects yet to be discovered. A good place to start looking for them is represented by the biasing effects the vocal tract could have on phonetics and phonology^12^. Other genes are known to have an effect on language and speech (e.g., *ROBO1, KIAA0319, CNTNAP2*, or *DCDC2* to name just a few) but we currently know too little about their functions and their variants across human populations to fruitfully speculate about their possible role in biasing particular directions in the cultural evolution of languages. Nevertheless, advances in understanding the genetic influences on language and speech, coupled with the availability of ancient DNA, may make it possible to speculate with more certainty about our ancestor's languages.

*Fourth*, if languages have much deeper historical roots than we have so far supposed, we need to find some way to extend the reach of historical linguistics. The comparative method, the classic way to demonstrate language relatedness, relies on lexical parallels or cognates whose signatures are steadily eroded by sound and meaning changes. Consequently, most linguists believe that the maximum reconstructed time-depth is about 10,000 years. Dunn et al. (2005, 2008) showed that structural features of language can effectively mirror the information in the vocabulary, and may potentially reach back 10,000 years or more where cognates have been lost. The method presumes that on average structural properties of languages change less often than words, and this is probably true: When changes of structure are reconstructed across the whole tree for large language families, we find that individual structural features (like major word order changes) change on average within a lineage just once in many thousands of years (Dunn et al., 2011). But whether hand-picked core vocabulary (like the Swadesh list or the new Leipzig-Jakarta list) changes faster than structure in general is still controversial (Greenhill et al., 2010). What one may hope is that some combinations of structural features will prove so conservative that they will allow deep reconstruction (Dediu, 2011). We have recently shown that, by combining structural features with information derived from other sources (in the form of language family trees) using Bayesian phylogenetic techniques, it is seemingly possible to pick up ancient signals of relatedness across the Bering Straits linking North-East Eurasian and American languages (Dediu and Levinson, 2012). Such links plausibly predate the loss of the land-bridge due to sea level rises c. 10,000 years ago, and similar analyses using structural data also point to Pleistocene language connections in Island Melanesia (Dunn et al., 2005) and Sahul (Reesink et al., 2009) predating the loss of other land bridges at the same time.

*Fifth*, the greater antiquity of language has important consequences for our theories of linguistic diversity. Traditionally it has been supposed that the current linguistic diversity (c. 7000 extant languages) offers a good basis for extrapolation of linguistic universals, or intrinsic constraints on linguistic capacity. But recent developments in the computational phylogenetics of language structure have revealed that structural change in languages is on average remarkably slow—on the order of tens of thousands of years (Dunn et al., 2008, 2011). On the standard picture modern humans left Africa in very small numbers not before, say, 70 kya, and perhaps as late as 50 kya. The genetic bottleneck that has been detected and dated to about this time (Amos and Hoffman, 2010) implies a cultural bottleneck—just a handful of languages accompanied the first migrants. If all the languages we currently have are the descendants of this small set (plus the stocks remaining in Africa), then the diversity we now have does not adequately sample the “design space” of possible languages at all (Evans and Levinson, 2009). After all, language families like Indo-European can be traced back ~9000 years (Atkinson and Gray, 2006; Pagel, 2009); so 6 or 7 such steps take us right back to the great diaspora. Our 7000 languages then tell us a story of historical relatedness and not much about the intrinsic limitations on the design space. The traditional goals of language typology, namely discovering language universals, would then be misguided—the data would tell us very little interesting about intrinsic constraints on possible languages.

However, some language families and linguistic features are very conservative (Dediu, 2011; Dunn et al., 2011) and such slow rates of change seem unable to account for the current diversity evolving since the expansions out of Africa. But if modern humans exiting from Africa interacted and interbred with Neandertals (and later, on their way through Asia, with Denisovans), then their contribution, we propose, might have shaped modern linguistic diversity. Neandertal (and Denisovan) languages would have offered a reservoir of linguistic diversity, on which the ancestors of our 7000 current languages may have drawn. Then the present-day languages would, to some extent, sample a wider part of the possibility space for languages, drawing on the Neandertal exploitation of that space over half a million years. At the same time, we have to remember that those ancestors of modern humans who continued to inhabit Africa also enjoyed half a million years of linguistic evolution^13^.

As we have seen, the two human lineages probably interacted, interbred, and borrowed culture. There were numerous points and periods of contact, e.g., early in the Near East around 100 kya, and thereafter at different locations in Eurasia. Although material culture suggests this interaction may have been mostly unidirectional, from modern humans to Neandertal groups, Neandertals no doubt had many useful concepts and techniques for exploiting their boreal habitats. Assuming again, as we have argued, that the two groups had similar speech and languages, four speculative scenarios can be imagined:

### Scenario 1

#### Language shift: modern humans adopted Neandertal languages

This is not likely, since in general the bearers of superior technology get aped and not the other way around. However, there are exceptions, for example the adoption by previously Indo-European farmers of a Saami language spoken by hunter-gatherers, namely Finnish (Sajantila et al., 1996). If this happened, there should be a radical discontinuity between the languages of Africa and the languages of the rest of the world, and no such discontinuity has yet been found (Cysouw and Comrie, 2009).

### Scenario 2

#### Language extinction: Neandertals interacted little and when they did they adopted modern human languages

On this scenario the technologically superior and demographically more numerous modern humans simply swamped Neandertals' languages and culture. In this case there should be no particular differences between African languages and those of the rest of the world, and there would have been limited time depth for linguistic diversification and exploration of the possibility space for languages as discussed above.

### Scenario 3

#### Pidginization: a new type of language is born by radical simplification

Pidginization is associated with colonization and rapid expansion of trade networks. Two human groups, in symmetrical relation, find a radical new solution to coexistence, dismantling two languages and rebuilding a third from the bits. This scenario does not seem consistent with the low level of contact in hunter-gatherer groups, nor with the archaeological record, which shows imported tool types in the Neandertal sites but not a radical rebuild of existing assemblages.

### Scenario 4

#### Sustained low intensity contact: a moderate exchange of lexicon and structure

This scenario is the most likely in our view. The two lineages would have been in protracted contact (as they were in the Middle East for up to 50,000 years). Technological and material exchange was mostly from modern humans to Neandertals, and the language borrowings may have followed suit. But material culture and language often part company—in Melanesia, for example, the technology and most of the material culture is uniform in both those societies speaking the more recently introduced Austronesian languages and those speaking the indigenous Papuan languages. It is quite probable that the Neandertals had both material and immaterial cultural tricks of considerable value for cultural adaptation to the new non-African environments (as they presumably did on the genetic side; Hawks and Cochran, 2006), and that these may have induced linguistic loans along with language structure.

Evidence related to these different scenarios comes from the archaeological data already mentioned, which points to, e.g., extensive overlap between Neandertal and modern human populations in Northern Europe, with consequent borrowing of material culture (Hublin et al., 2012). But a more directly linguistic way to test these scenarios is to look for subtle structural differences between the languages of Africa and the rest of the world, as any such finding might point to remnants of Neandertal languages. For example, we could compare structural profiles of languages in Africa and outside it using pattern detection techniques such as Support Vector Machines, or by looking at the distribution of structural differences in geographic space. Similarly, differences between Papuan and Australian languages, on one hand, and the other languages on the other, might offer a glimpse of an outcome of the interaction between human and Denisovan languages: recently, David Gil (2011) suggested that features related to lower grammatical complexity present in the languages of the “Mekong-Mamberamo” linguistic area (from mainland SE Asia through most of Indonesia and into the western half of New Guinea) could be a remnant of contact with Denisovans. This suggestion is based on McWhorter's (2008) speculation that the simplification of the Austronesian languages on the island of Flores could be due to early contact with *H. floresiensis*. Realistic computer models of these particular encounters might help us better quantify what are the most probable consequences for today's languages and provide clear and testable predictions of these hypotheses. Finally, using the rates of structural change mentioned above, it is in principle possible to construct forward models that attempt to generate current linguistic diversity within the c. 60 k years since the last great exodus of modern humans from Africa, and to test whether it is necessary to draw on ancient reservoirs of linguistic diversity already present in Eurasia.

## Conclusions

In this paper, we have tried to review the evidence supporting the claim that Neandertals, Denisovans and contemporary modern humans shared a similar capacity for modern language, speech and culture. Furthermore, we argued that regarding these lineages as different species is unhelpful, and that their admixture probably shaped present-day genetic and linguistic diversities. Moreover, we propose an approach which might allow us to increase the focus of scientific inquiry into the deep past of linguistic diversity, by comparing present-day African and non-African (and possibly Papuan and Australian to non-Papuan non-Australian) structural linguistic distributions. We need to fully grasp the implications of the fact that human evolution (ancient, recent and current) is a reticulated process, which has the consequence, among others, that we have to regard language as a very old cultural evolutionary process in which both vertical and horizontal processes are essential contributors. On this background of shared capacities, understanding the relatively small differences between modern humans, Neandertals and Denisovans will help shed more light on the nature and evolution of speech and language.

The antiquity of modern language and speech capacities, going back to at least the last common ancestor of Neandertals, Denisovans and modern humans some half a million years ago, raises new and interesting questions concerning the nature of the linguistic design space, the relationship between biological and cultural evolution, and the time frame for the emergence of modern human traits, and language in particular.

### Conflict of interest statement

The authors declare that the research was conducted in the absence of any commercial or financial relationships that could be construed as a potential conflict of interest.

## Footnotes

^1^Consequently, we use the term “human” to include the sister lineages (Neadertals and Densiovans) modern humans interbred with; see below for discussion of the species concepts involved.

^2^For discussion of unclarities concerning the distinction FLN vs. FLB see, e.g., Jackendoff and Pinker (2005).

^3^We avoid binomial species names (i.e., *H. sapiens* and *H. neanderthalensis*) at this level on purpose, as they implicitly assume species-grade differences.

^4^Here we gloss over the current nomenclatural controversy concerning *H. heidelbergensis* (e.g., Hublin, 2009): the Atapuerca fossils are at least in a lineage close to this species. We are thus using *H. heidelbergensis* as the name of the most recent common ancestor of Neandertals, Denisovans and modern humans, attributing by default to this ancestor whatever can be shown to be common across the three lineages.

^5^It is important to highlight how much we do not yet know about human genetic variation: recently Mendez et al. (2013a) reported the totally unexpected fortuitous discovery of a Y chromosome in a living African American so divergent that its most recent common ancestor with the other Y chromosomes must be pushed back to 237–581 kya, older than the fossil evidence for modern humans.

^6^However, it is unclear what exactly this aminoacid change in the Laminin G-like domain of the CASPR protein (the product of *CNTNAP2*) does. This results in the replacement of the primitive (non-human primates, Neandertals and Denisovans) isoleucine by the derived (modern human) valine, but these aminoacids have similar properties, and valine is also present in for example mice and rats (Cuadrado et al., 2013).

^7^The most recent certain date for Neandertals in Europe comes from Gibralter, with a radiocarbon date of 28 kya, but they may have hung on longer (Finlayson et al., 2006). Using more refined dating techniques, all these dates may be upward revised (Wood et al., 2013), but they continue to imply some considerable overlap of Neandertals and modern humans in the west, with more extended overlap in eastern Eurasia.

^8^A reviewer queries whether genuine hierarchy exists in action sequences, suggesting that these can be reduced to a TOTE structure (Miller et al., 1960). But as soon as one subroutine calls another, as in the case of complex tool manufacture, genuine hierarchy is involved. See also Levinson (2013).

^9^A reviewer remarked that “no one has ever disagreed” that language evolved under natural selection, but in fact Chomsky and colleagues have systematically done so. See for example Chomsky (1972: 97; 1988: 170, 183) and Berwick (1998: 322).

^10^This perspective is entirely compatible, pace a reviewer, with recent developments in generative theory where variation is relegated to peripheral processes.

^11^Only the ancestral allele of *Microcephalin* was found in the single individual genotyped by Lari et al. (2010) and also seems to be present in the individuals used by Green et al. (2010) for the draft Neandertal genome. The derived allele of *ASPM* is estimated as far too young (5800 years, 95% confidence interval 500–14100; Mekel-Bobrov et al., 2005) to have been present in Neandertals.

^12^This direction is currently investigated by a project led by the first author and there are already hints that there is intra- and inter-populations variation in components of the vocal tract (e.g., the hard palate) at least partly under genetic influences, and that this variation might indeed bias speech.

^13^These ideas need rigorous testing through computer models as it is not at all clear how these 500 kya of linguistic evolution play out in various demographic scenarios. Conceivably, a stable population of large size will produce a quite different pattern of linguistic diversity to a set of many small individually fleeting populations in contact.
